# Combined mutations of *ASXL1, CBL, FLT3, IDH1, IDH2, JAK2, KRAS, NPM1, NRAS, RUNX1, TET2 *and *WT1 *genes in myelodysplastic syndromes and acute myeloid leukemias

**DOI:** 10.1186/1471-2407-10-401

**Published:** 2010-08-02

**Authors:** Julien Rocquain, Nadine Carbuccia, Virginie Trouplin, Stéphane Raynaud, Anne Murati, Meyer Nezri, Zoulika Tadrist, Sylviane Olschwang, Norbert Vey, Daniel Birnbaum, Véronique Gelsi-Boyer, Marie-Joelle Mozziconacci

**Affiliations:** 1 Centre de Recherche en Cancérologie de Marseille; Laboratoire d'Oncologie Moléculaire; UMR891 Inserm; Institut Paoli-Calmettes; Marseille, France; 2 Département de BioPathologie, Institut Paoli-Calmettes, Marseille, France; 3 Service de Médecine Interne, Centre Hospitalier Général, Martigues, France; 4 Service de Médecine Interne-Oncologie, Hôpital de Salon-de-Provence, Salon-de-Provence, France; 5 Faculté de Médecine, Université de la Méditerranée, Marseille, France; 6 Département d'Hématologie, Institut Paoli-Calmettes, Marseille, France

## Abstract

**Background:**

Gene mutation is an important mechanism of myeloid leukemogenesis. However, the number and combination of gene mutated in myeloid malignancies is still a matter of investigation.

**Methods:**

We searched for mutations in the *ASXL1, CBL, FLT3, IDH1, IDH2, JAK2, KRAS, NPM1, NRAS, RUNX1, TET2 *and *WT1 *genes in 65 myelodysplastic syndromes (MDSs) and 64 acute myeloid leukemias (AMLs) without balanced translocation or complex karyotype.

**Results:**

Mutations in *ASXL1 *and *CBL *were frequent in refractory anemia with excess of blasts. Mutations in *TET2 *occurred with similar frequency in MDSs and AMLs and associated equally with either *ASXL1 *or *NPM1 *mutations. Mutations of *RUNX1 *were mutually exclusive with *TET2 *and combined with *ASXL1 *but not with *NPM1*. Mutations in *FLT3 (*mutation and internal tandem duplication), *IDH1*, *IDH2*, *NPM1 *and *WT1 *occurred primarily in AMLs.

**Conclusion:**

Only 14% MDSs but half AMLs had at least two mutations in the genes studied. Based on the observed combinations and exclusions we classified the 12 genes into four classes and propose a highly speculative model that at least a mutation in one of each class is necessary for developing AML with simple or normal karyotype.

## Background

Myeloid leukemogenesis is a complex process that transforms a regulated hematopoietic stem or progenitor cell into a proliferative cell unable to differentiate. Several genetic alterations such as translocations, gene mutations and deletions play a role in this process. Balanced translocations generating gene fusion play a major leukemogenic role in some classes of leukemias. However, they are rare in myelodysplastic syndromes (MDSs) and the majority of adult acute myeloid leukemias (AMLs) have a normal karyotype (NK-AMLs). MDSs are a heterogeneous group of clonal diseases characterized by bone marrow dysplasia, various degrees of cytopenia and a risk of progression to AML [1]. The alterations that lead to these pre-leukemic disorders are poorly defined [2]. Several genes have been identified recently as mutated in MDSs and AMLs, including *ASXL1*, located in chromosome arm 20q, *CBL*, located in 11q, and *TET2*, located in 4q [3-11]. These mutations are associated with biological and prognostic features [9,11].

Similarly, gene mutations are likely to play a role in the development of NK-AMLs. Indeed, the identification of mutations in several genes such as *NPM1 *and *FLT3 *has revealed prognosis subgroups and modified the clinical management of these leukemias [12,13]. A recent whole-genome sequencing study of an AML case has revealed mutations in *IDH1*, which encodes the enzyme isocitrate dehydrogenase [14]. AMLs occur *de novo *or after a chronic phase (hereafter called primary and secondary, respectively). Such chronic phase can be an MDS. We have recently shown that mutations in *ASXL1*, in contrast to *NPM1 *mutations, are found in MDSs and in secondary AMLs [15].

Mutation data are in line with the multi-hit model proposed by Gilliland suggesting that many gene mutations play a role in leukemogenesis [16]. Now that more and more gene mutations are reported it is important to determine when they occur in the various types of malignant hematopoietic diseases and how they combine in the development of leukemia. To begin to answer these questions we searched for mutations in twelve selected genes in a panel of 129 MDS and AML samples.

## Methods

### Patients and samples

All patients signed an informed consent and the study was approved by the institutional review board ("Commission d'Orientation Scientifique") of Institut Paoli-Calmettes.

The 129 samples were selected on the absence of known balanced translocation, and in the absence of complex karyotype for the AML series (as defined by more than three alterations). They included 65 cases of MDS (Additional file 1**Table S1**), comprising, according to the WHO criteria [17], 5 refractory anemia (RA), 13 refractory anemia with ring sideroblasts (RARS) (including one with myelofibrosis), 7 refractory cytopenia with multilineage dysplasia (RCMD), 16 refractory anemia with excess of blasts type 1 (RAEB1), 19 refractory anemia with excess of blasts type 2 (RAEB2) and 5 MDS-unclassified (MDS-U) cases. Six cases were secondary to hematopoietic or non-hematopoietic diseases (na in IPSS column, Additional file 2** Table S1**). The majority of MDS samples were collected at the time of diagnosis; some were in therapeutic abstention of a known MDS and some were under symptomatic treatment. Seventeen cases were IPPS low risk (0), 23 were int-1 (0.5-1), 12 were int-2 (1.5-2) and 7 were high risk (≥2.5).

We studied 64 AMLs (Additional file 2** Table S2**) including 46 primary cases and 18 transformations of a previous myeloid disease (the same cohort of 64 AML patients, listed in Additional file 2** Table S2**, had been analyzed for mutations in the *NPM1, FLT3, CEBPA, NRAS, KRAS, JAK2*, and *ASXL1 *genes and the results already reported in ref. 15). The panel comprised 47 cases of NK-AML and 17 cases with either trisomy 8 (n = 14), 9q deletion (HD-0632), trisomy 11 (HD-0304) or 20q deletion (HD-0381) as a sole karyotypic abnormality.

Array-comparative genomic hybridization (aCGH) had been performed on almost all samples and had allowed the detection of deletions and breaks [9,10,15,18,19].

### DNA sequencing

DNA sequencing of exon-coding sequences of *ASXL1, CBL, FLT3, IDH1, IDH2, JAK2, KRAS, NPM1, NRAS, RUNX1, TET2 *and *WT1 *was done as follows. PCR amplifications of bone marrow cell DNA were done in a total volume of 25 μl PCR mix containing at least 5 ng template DNA, Taq buffer, 200 μmol of each deoxynucleotide triphosphate, 20 pmol of each primer and 1 unit of Hot Star Taq (Qiagen). PCR amplification conditions were as follows: 95°C 10 min; 95°C 30 sec, 55°C 30 sec, 72°C 30 sec to 1 min depending on PCR product length for 35 cycles; 72°C 10 min. PCR products were purified using Millipore plate MSNU030. One microliter of the purified PCR products was used for sequencing using the Big Dye terminator v1.1 kit (Applied Biosystems) including the forward or reverse primer. After G50 purification, sequences were loaded on an ABI 3130XL automat (Applied Biosystems). The sequence data files were analyzed using both SeqScape and Phred/Phrap/Consed softwares and all mutations were confirmed on an independent PCR product. The exons studied were as follows: *ASXL1 *exon 12, *CBL *exons 8 and 9, *FLT3 *exons 14, 15 and 20, *IDH *exon 4, *JAK2 *exon 14, *RAS *exons 1 and 2, *NPM1 *exon 12, *RUNX1 *exons 1 to 8, *TET2 *exons 3 to 11, *WT1 *exons 7 and 9. The primers for sequencing are listed in [Additional file 3].

## Results

### Mutations in MDSs

We had previously shown that *ASXL1 *frameshift and nonsense mutations occurred in exon 12 [10]. *ASXL1 *exon 12 frameshift mutations (10 times the same p.Gly646Trpfsx12) were observed in 12 out of the 65 MDS cases (18.5%) including 1 out of 5 RA (20%), 2 out of 16 RAEB1 (12.5%) and 9 out of 19 RAEB2 (47.4%) (Additional file 1** Table S1**). We found 12 cases with *TET2 *mutation (18.5%) and 4 with *RUNX1 *mutation (6.2%). One patient (HD-0311) had two *TET2 *mutations. *TET2 *mutations were frequent in RAEB1 (7/16, 43.8%). Mutations in *RUNX1 *and *TET2 *were mutually exclusive but both could associate with *ASXL1 *mutations: two cases showed both an *ASXL1 *and a *TET2 *mutation and three cases both an *ASXL1 *and a *RUNX1 *mutation. One case of *ASXL1 *deletion (HD-0190) and one case of *TET2 *deletion (HD-0145) have been reported [9,10]. One case (HD-0232) had a break in *RUNX1 *detected by aCGH (not shown).

**Figure 1:**
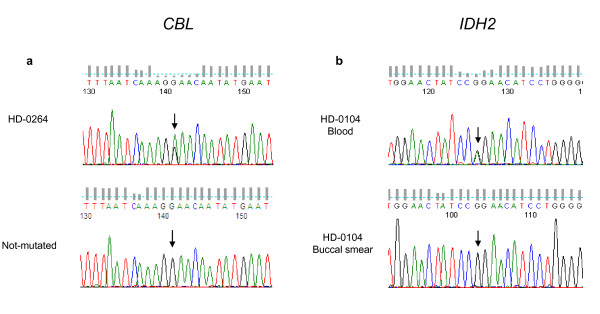
**Examples of mutations in leukemia target genes**. a. sequence of *CBL *demonstrating c.1096G>A base substitution in the forward sequence at the position indicated by an arrow in HD-0264 patient with trisomy 11. The mutation leads to Glu366Lys change in the protein (not tolerated, SIFT). Sequence numbering is according to Genbank accessions numbers NM_005188 and NP_005179. The same sequence from another patient without mutation is shown below for comparison. b. Novel IDH2 mutation in MDS and AML patients. Sequence of *IDH2*, demonstrating c.419G>A base substitution in the reverse sequence at the position indicated by an arrow in blood cells of patient HD-0104 and its absence in the patient's buccal smear. The mutation leads to Arg140Gln (R140Q) change in the protein (not tolerated, SIFT). Sequence numbering is according to Genbank accessions numbers NM_002168.2 and NP_002159.2.

We did not find any *FLT3, NPM1 *or *WT1 *mutation. One MDS-U had a *JAK2 *mutation and one RCMD case a *KRAS *mutation. Five cases, all RAEB2, were mutated in *CBL*. In one of these the mutation was homozygous. One subtitution occurred in the case with trisomy 11 (HD-0264), and showed a 2/3 ratio with the wild-type residue, suggesting that the mutated allele was duplicated (Figure 1a). This is in agreement with a potential gain-of-function of the mutated CBL [20]. We found 5 IDH mutations in the 65 cases (7.7%), including 2 mutations in *IDH1 *and 3 in *IDH2*.

### Mutations in AMLs

We found heterozygous nonsense or frameshift mutations of *ASXL1 *in 11 out of the 64 cases and one case (HD-0381) was known to have a deletion (Additional file 2** Table S2**) [15]. As previously shown [15]*NPM1 *mutations were found in 28 cases, mostly primary cases. We found 9 *TET2 *mutations in 9 cases, and 10 *RUNX1 *mutations in 9 cases (HD-0790 had two *RUNX1 *mutations). In addition, HD-0198 had a break in *RUNX1 *[19]. These alterations were found in both primary and secondary AMLs. Mutations in *RUNX1 *and *TET2*, *RUNX1 *and *NPM1*, and *ASXL1 *and *NPM1 *were mutually exclusive, respectively. *TET2 *mutations could associate with either mutated *NPM1 *or *ASXL1*.

Nineteen cases (mostly primary) had *FLT3 *internal tandem duplication or point mutation. Three cases had a *RAS *mutation and one a JAK2 V617F mutation. The only *CBL *mutation was found in the case with a trisomy 11 (HD-0304, the corresponding transformed state of HD-0264 MDS). Three primary cases (6.5%) showed a *WT1 *mutation. Three cases were mutated in *IDH1*. Four cases showed the Arg172Lys mutation in IDH2. We also found 11 cases (17%) with the Arg140Gln substitution in IDH2 (Figure 1b).

### Paired cases

For two patients both the chronic (HD-0173, HD-0264) and acute (HD-0790, HD-0304) phases of the disease were available (in bold in Tables). RARS HD-0173 had a mutated *RUNX1 *allele. The corresponding AML (HD-0790) had two *RUNX1 *mutations. RAEB2 HD-0264 had a mutation in *ASXL1, CBL *and *TET2 *genes. In the corresponding AML we did not identify any additional mutation in the studied genes.

### Summary of results

Additional file 4** Table S3 **shows a summary of the results on mutated (cases with two mutations are counted as one) and deleted/broken cases. Mutations of *ASXL1 *were frequent in MDSs and secondary AMLs. Mutations of *TET2 *occurred with a similar frequency in MDSs and AMLs. *ASXL1 *and *CBL *mutations were associated with chronic phase, mainly RAEB2. Overall, advanced stage MDSs had more mutations. *NPM1*, *FLT3 *and *WT1 *mutations were associated with primary AMLs. Mutations of *IDH1/2 *were rare in chronic phase. Not surprisingly, the number of mutated genes per case was lower in MDSs than in AMLs: only 14% MDSs but more than half AMLs had at least two gene mutations.

## Discussion

We searched for mutations in a series of selected genes. Mutations in some of these genes, such as *ASXL1, CBL, IDH *and *TET2*, have been identified only recently in myeloid diseases and have never been surveyed together to date. A number of issues need be discussed.

The frequencies of mutations we observed are close to what has been reported individually for each gene so far. This is true for example for *IDH1*, *NPM1*, *TET2 *and *WT1 *[1,3-9,14,21,22]. In MDS we found slightly more mutations of *CBL *than previously reported [11]. The IDH2 Arg140Gln was frequent in AMLs, rare in MDSs. The mutation was not present in the buccal smear DNA of a patient with AML (HD-0104), showing it was acquired. The Arg140Gln mutation in IDH2 has been reported in recent studies of myeloid diseases [23,24]. A recent study has described *ASXL1 *as the most frequently mutated gene in advanced MDSs [25], which we confirmed here.

### A repertoire of mutations

Based on the known functions of the proteins, on a previous model and classification [16], on where the mutations were present (MDSs and/or secondary AMLs and/or primary AMLs) and on how they combined (mutual exclusion or association), we tentatively grouped the genes in four classes. The first class includes *RUNX1 *and *TET2*. Mutations in these genes may cause clonal dominance of hematopoietic stem cells [3]. *ASXL1 *and *NPM1 *would constitute class II. Mutations in these genes may promote a pathway leading towards either primary or secondary AML [15]. Genes associated with signaling pathways and proliferation [16] (*CBL, FLT3, JAK2, RAS*) define class III. *JAK2 *mutation plays little role in MDSs and NK-AMLs. We have shown that FLT3 signaling is regulated by CBL [26]. However, the two alterations may not be equivalent; if in our study mutations in these genes were mutually exclusive and were both more frequent in AMLs than in MDSs, *CBL *but not *FLT3 *mutations were frequent in RAEB2 cases. This is in agreement with recent studies that showed alterations of *CBL *in chronic myeloid diseases [11,27]. Finally, for three reasons we grouped *IDH1*, *IDH2*, and *WT1 *in a putative class IV. First, *IDH *and *WT1 *mutations were exclusive but could co-occur with mutations in genes from other classes. Second, they occurred primarily in AMLs and were rare in MDSs. Third, mutations of these genes could be associated with modifications of the HIF1 and oxygen-sensing pathways [28,29]. Class IV mutations are rather associated with acute phase.

**Figure 2:**
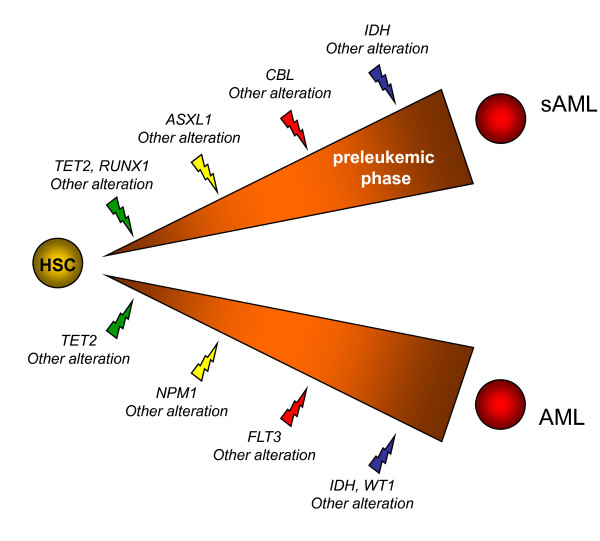
**Hypothetical model summarizing frequent gene mutations in the development of two types of acute myeloid leukemia (AML)**. A hematopoietic stem cell (HSC) or a progenitor cell acquires a series of mutations beginning in class I genes (green). Different additional events (yellow, red and blue) induce clonal expansion and differentiation block and lead either towards secondary AML (sAML) via a patent preleukemic phase or AML without patent preleukemic phase. The order of events may vary.

The existence of many cases with few or no mutations in the selected genes, especially in some classes of MDSs such as MDS-U, RA, RARS and RCMD but also in primary AMLs (half of the cases with no or one mutation), suggests that other genes remain to be studied or discovered. Indeed, our study did not include several target genes, such as *CBFB *[30], *CDKN1A/B*, *CEBPA*, *ETV6*, *KIT*, *NF1*, *NFIA *[31], *P53, RB1 *and *UTX *[32]; it did not include either a search for the recently discovered mutations of the *EZH2 *gene [33,34]. Moreover, our aCGH analyses of the same series showed that some cases without mutation in the studied genes do have deletions of other cancer genes (for example *CDKN1B, ETV6 *and *UTX *[32] are deleted in HD-0205) [9,10,15]. Thus, the repertoire of altered genes in both MDSs and AMLs is likely to include many genes and it is probable that whole-genome sequencing studies will confirm this. Because of this, and although no case had four mutations, we propose that AML develops following - at least - four cooperating mutations, one from each class (Figure 2). This is highly speculative however; the identification of new target genes and the study of others will lead to a more precise picture. Also, because we did not study AMLs with balanced translocation or complex karyotype this model is proposed only for AML with simple or normal karyotype.

### Random or ordered accumulation?

An important question is whether the mutations occur in a necessary order or randomly until differentiation of the malignant hematopoietic clone is completely blocked. RARS HD-0173 (one *RUNX1 *mutation) and AML HD-0790 (two *RUNX1 *mutations) are instructive samples from the same patient. It is tempting to interpret this observation as a bi-allelic inactivation of the *RUNX1 *gene. *RUNX1 *mutations are frequent in chronic myelomonocytic leukemia [19] but rare in myeloproliferative neoplasms except at the acute phase [35]. In our series, mutations in *TET2 *were equally frequent in MDSs and AMLs. Taken together this suggests that inactivation of class I tumor suppressors (*RUNX1, TET2*) can intervene at any stage, i.e. early or at progression. In studies of AMLs secondary to myeloproliferative neoplasms (MPN) mutations of *TET2 *have indeed been shown to occur early or late [30,36]. A recent study showed that *ASXL1 *mutations were present at the chronic stage and, in agreement with our findings, that *ASXL1*, *JAK2 *and *TET2 *have non overlapping contributions to myeloid transformation [36]. The order of occurrence of *ASXL1*, *TET2 *and *RUNX1 *mutations may vary in different diseases (MPNs and MDSs) and in different patients. However, the number of paired cases was much too low in our series to draw any firm conclusion. The study of more paired cases is necessary to definitely clear this issue. In contrast, some mutations such as *IDH1 *and *IDH2 *amino acid changes were detected at the AML rather than the MDS stage. Mutations in proliferation genes - except in *CBL *- were rare in MDS. Taken together this suggests that mutations in classes III and IV may occur late in leukemogenesis.

### Myeloid malignancies and cancer genes

The study of myeloid malignancies may modify the classical view of cancer gene inactivation. *IDH1 *mutations do not result in a loss but in a change in activity [37]. This is in agreement with the fact that, in contrast to *ASXL1, RUNX1 *and *TET2*, we never observed break or deletion at *IDH *loci by aCGH analysis. *WT1 *is mutated but also overexpressed in AMLs. Mutations in *CBL *are associated with a gain-of-function [20]. *NPM1 *overexpression leads to increased cell growth [38]. We have not so far found two mutations (or mutation and deletion) in *ASXL1 *in the same sample. It is possible that *ASXL1 *mutations are also associated with a gain-of-function [39]. Only *RUNX1 *and *TET2 *may be inactivated in the two-hit fashion that corresponds to "classical" tumor suppressors.

### Impact of mutations on prognosis

Due to relatively small numbers our purpose was not to address prognosis issues. Besides, it may be illusory to study prognostic impact of tumor suppressors in the absence of knowledge of yet unknown genes that may serve the same function. However, some features could be briefly noted. In contrast to *TET2 *mutations, which were relatively evenly distributed among IPSS classes, *ASXL1*, *CBL *and *IDH *mutations were associated with more aggressive MDSs. Lower risk MDSs had fewer mutations of these genes than higher risk MDSs. Half of the 12 MDS with int-2 IPSS showed *ASXL1 *mutations, and reciprocally half of the *ASXL1 *mutations were in int-2 cases. Four *CBL *mutations were found in int-2 cases. The 7 MDSs with high IPSS had an abnormal karyotype but few mutations (*ASXL1 *and *RUNX1 *in HD-0377, *CBL *in HD-0193); this is not surprising since karyotype status has an important weight on IPSS as one of its components. A long follow-up on disease evolution should tell if and how the mutational status impacts on MDS progression to AML and should be used to fine-tune IPSS.

## Conclusion

We have reported here the first comprehensive study including recently discovered gene mutations in MDSs and AMLs. We have proposed a speculative model of cooperative leukemogenesis for AML with simple or normal karyotype, which needs to be confirmed by the study of more cases and completed by the discovery of more genes. As this it represents a step towards the necessary determination of the complete mutational status of myeloid malignant diseases.

## Competing interests

The authors declare that they have no competing interests.

## Authors' contributions

JR, NC, VT, SR and SO obtained and analyzed gene sequencing data. AM, MN, ZT, VGB; MJM and NV provided samples and bioclinical data. NV, DB, VGB and MJM designed and supervised the study, analyzed the data and wrote the manuscript. All authors read and approved the final manuscript.

## Pre-publication history

The pre-publication history for this paper can be accessed here:

http://www.biomedcentral.com/1471-2407/10/401/prepub

## Supplementary Material

Additional file 1 **Table S1 Mutations of candidate genes in a series of myelodysplastic syndromes**. Click here for file

Additional file 2 **Table S2 Mutations of candidate genes in a series of AMLs**. Click here for file

Additional file 3 **Oligonucleotide primers used for sequencing the coding regions of the selected genes**. Click here for file

Additional file 4 Table S3 Summary of results. Click here for file
