# Supplementary material for: Combined mutations of ASXL1, CBL, FLT3, IDH1, IDH2, JAK2, KRAS, NPM1, NRAS, RUNX1, TET2 and WT1 genes in myelodysplastic syndromes and acute myeloid leukemias
Source: BMC Cancer. 2010 Aug 2;10:401. doi: 10.1186/1471-2407-10-401 (PMC2923633; doi:10.1186/1471-2407-10-401)
Supplement: Additional file 1 — Table S1 Mutations of candidate genes in a series of myelodysplastic syndromes. [file 1471-2407-10-401-S1.PDF]

Number	Sex/age	WHO subtype	IPSS	RUNX1 (exons 1-8)	TET2 (exons 3-11)	ASXL1 (exon 12)	NPM1 (exon 12)	CBL (exons 8, 9)	FLT3 ITD/TKD	JAK2 (V617F)	RAS (exons 1,2)	IDH1 (exon 4)	IDH2 (exon 4)	WT1 (exons 7, 9)	Number of altered alleles	Karyotype
HD-0288	M/61	RA	0	no	no	no	no	no	no	no	no	no	no	no	0	46,XY[20]
HD-0292	M/76	RA	0	no	no	no	no	no	no	no	no	no	no	no	0	46,XY[20]
HD-0302	M/75	RA	0,5	no	no	no	no	no	no	no	no	no	no	no	0	46,XY[20]
HD-0378	M/68	RA	1	no	no	no	no	no	no	no	no	no	no	no	0	46,XY,del(20)(q11q13)[20]
HD-0384	F/84	RA	0	no	no	p.Ser1457ProfsX18	no	no	no	no	no	no	no	no	1	46,XX,del(5)(q13q31)[10]/46,XX[10]
HD-0145	F/68	RARS	0,5	no	deletion	no	no	no	no	no	no	no	no	no	1	46,XX[20]
HD-0150	F/83	RARS	0	no	no	no	no	no	no	no	no	no	no	no	0	46,XX[5]
HD-0165	M/60	RARS	0	no	no	no	no	no	no	no	no	no	no	no	0	46,XY[20]
**HD-0173**	M/71	RARS	0	p.Asn146MetfsX6	no	no	no	no	no	no	no	no	no	no	1	46,XY[20]
HD-0192	M/82	RARS	0,5	no	no	no	no	no	no	no	no	no	no	no	0	46,XY,inv(9)(p12q12)?c[20]
HD-0246	M/80	RARS	0	no	no	no	no	no	no	no	no	no	no	no	0	45,X,-Y[17]/46,XY[5]
HD-0258	M/81	RARS	0,5	no	no	no	no	no	no	no	no	no	no	no	0	47,XY,+8[10]/46,XY[10]
HD-0260	F/57	RARS	0,5	no	no	no	no	no	no	no	no	no	no	no	0	46,XX[20]
HD-0262	F/73	RARS	0	no	p.Gln1524X	no	no	no	no	no	no	no	no	no	1	46,XX[20]
HD-0285	F/87	RARS	0	no	no	no	na	no	no	no	no	no	no	no	0	46,XX[20]
HD-0430	F/68	RARS	0	no	no	no	no	no	no	no	no	no	no	no	0	46,XX[20]
HD-0487	M/70	RARS	0	no	no	no	no	no	no	no	no	no	no	no	0	46,XY[20]
HD-0637	M/64	RARS+MF	0	no	p.Leu1360Gln	no	no	no	no	no	no	no	no	no	1	46,XY[20]
HD-0172	M/79	RCMD	0,5	no	no	no	no	no	no	no	no	no	no	no	0	46,XY[20]
HD-0191	F/81	RCMD	0,5	no	no	no	no	no	no	no	no	no	no	no	0	46,XX[20]
HD-0235	F/51	RCMD	0	no	no	no	no	no	no	no	no	no	no	no	0	46,XX[20]
HD-0289	F/83	RCMD	0,5	no	p.Gln1627IlefsX31	no	no	no	no	no	no	no	no	no	1	46,XX[20]
HD-0371	M/74	RCMD	0,5	no	no	no	no	no	no	no	no	no	no	no	0	46,XY[20]
HD-0670	F/71	RCMD	0,5	no	no	no	no	no	no	no	p.Gly12Asp	no	no	no	1	46,XX[20]
HD-0747	M/77	RCMD	0,5	no	no	no	no	no	no	no	no	no	no	no	0	46,XY[20]
HD-0152	F/75	RAEB1	1	no	p.val218TrpfsX32	no	no	no	no	no	no	no	no	no	1	46,XX[20]
HD-0167	F/77	RAEB1	2,5	no	no	no	no	no	no	no	no	no	no	no	0	46,XX,-5,-6,-7,-17,+4mar[5]/46,XX[15]
HD-0183	M/70	RAEB1	0,5	no	no	no	no	no	no	no	no	no	no	no	0	46,XY[20]
HD-0190	M/77	RAEB1	2,5	no	no	deletion	no	no	no	no	no	no	no	no	1	44,XY,ins(2;12)(p23;q13q24),del(2)(q23q25),der(7;16)(p10;q10),-12,-18,del(20)(q11q13),+ mar[2]/47,sl,+1,-13,+3,mar[3]/43,sl,dup(1)(q25q32),del(7)(p11),-13[14]/46,XY[1]
HD-0196	M/78	RAEB1	0,5	no	p.Pro1419Arg	p.Gly646TrpfsX12	no	no	no	no	no	no	no	no	2	46,XY[20]
HD-0205	M/60	RAEB1	2,5	no	no	no	no	no	no	no	no	no	no	no	0	45,XY,del(4)(q?q?),del(5)(q22q34),-7,+8,-12,dup(12)(q21q24),-21,+mar[11]/46,XY[1]
HD-0233	M/62	RAEB1	0,5	no	p.Arg1440ThrfsX38	no	no	no	no	no	no	no	no	no	1	46,XY[20]
HD-0239	F/59	RAEB1	0,5	no	no	no	no	no	no	no	no	no	no	no	0	46,XX[20]
HD-0269	M/60	RAEB1	na	no	no	p.Gly646TrpfsX12	no	no	no	no	no	p.Arg132Cys	no	no	2	46,XY[20]
HD-0270	M/79	RAEB1	0,5	no	no	no	no	no	no	no	no	no	no	no	0	46,XY,del(5)(q15q34)[8]/46,XY[12]
HD-0286	F/73	RAEB1	2	no	p.Pro1575PhefsX31	no	no	no	no	no	no	no	no	no	1	46,XX,-7,+mar[3]/46,XX[17]
HD-0296	M/62	RAEB1	0,5	no	no	no	no	no	no	no	no	p.Arg132Cys	no	no	1	46,XY[20]
HD-0300	M/72	RAEB1	0,5	no	p.Lys1197Arg (nc)	no	no	no	no	no	no	no	no	no	1	46,XY[4]
HD-0311	M/85	RAEB1	0,5	no	p,Ser1286Pro; p.Asp1384His	no	no	no	no	no	no	no	no	no	2	46,XY[20]
HD-0394	F/70	RAEB1	na	no	no	no	no	no	no	no	no	no	no	no	0	46,XX[20]
HD-0434	M/65	RAEB1	0,5	no	p.Phe797SerfsX16	no	no	no	no	no	no	no	no	no	1	46,XY[20]
HD-0169	F/62	RAEB2	1,5	no	no	no	no	no	no	no	no	no	no	no	0	46,XX[20]
HD-0175	M/71	RAEB2	2	no	no	no	no	no	no	no	no	no	p.Arg140Gln	no	1	46,XY[20]
HD-0180	M/81	RAEB2	2	no	no	p.Gly646TrpfsX12	no	p.Tyr368X	no	no	no	no	p.Arg140Gln	no	3	46,XY[20]
HD-0186	M/81	RAEB2	na	p.Gly170Arg	no	p.Gly646TrpfsX12	no	no	no	no	no	no	no	no	2	46,XY[20]
HD-0193	F/69	RAEB2	3,5	no	no	no	no	p.Cys381Arg	no	no	no	no	no	no	1	47,XX,del(5)(q23q34),+21[10]/46,idem,-7[7]/48,idem,+22[3]
HD-0195	M/72	RAEB2	2,5	no	no	no	no	no	no	no	no	no	no	no	0	47,XY,+ 8[20]
HD-0208	F/50	RAEB2	1,5	no	no	p.Gly646TrpfsX12	no	no	no	no	no	no	p.Arg140Gln	no	2	46,XX[20]
HD-0210	M/72	RAEB2	2	p.Arg201Gln	no	p.Gly646TrpfsX12	no	no	no	no	no	no	no	no	2	46,XY[20]
HD-0232	M/63	RAEB2	na	break	no	no	no	no	no	no	no	no	no	no	1	na
HD-0250	M/80	RAEB2	2	no	no	p.Gly646TrpfsX12	no	no	no	no	no	no	no	no	1	47,XY,+X?c[20]
HD-0263	M/67	RAEB2	2	no	no	no	no	p.Pro417Leu (hom)	no	no	no	no	no	no	1	47,XY,+8[19]/46,XY[1]
**HD-0264**	M/68	RAEB2	1,5	no	p.Leu862SerfsX6	p.Gly646TrpfsX12	no	p.Glu366Lys	no	no	no	no	no	no	3	47,XY,+11[20]
HD-0275	M/83	RAEB2	na	no	no	p.Gly646TrpfsX12	no	no	no	no	no	no	no	no	1	46,XY,-7,+mar[5]/47,sl,+?del(7)(q11q36)[4]/47,sdl,del(14)(q2?3q3?1)[6]/46,XY[5]
HD-0375	M/76	RAEB2	2	no	no	no	no	p.Leu380Pro	no	no	no	no	no	no	1	47,XY,+8[20]
HD-0377	F/86	RAEB2	3	p.Ala142GlyfsX2	no	p.Arg596ProfsX23	no	no	no	no	no	no	no	no	2	45,XX,-7[14]/46,XX[6]
HD-0411	F/59	RAEB2	3	no	no	no	no	no	no	no	no	no	no	no	0	46,XX,del(5)(q15q35)[16]/46,XX,t(14)(q10)[3]/46,idem,del(7)(q11q35-36)[3]/46,XX [1]
HD-0425	M/88	RAEB2	1,5	no	no	p.Gly646TrpfsX12	no	no	no	no	no	no	no	no	1	46,XY[20]
HD-0486	F/58	RAEB2	1	no	no	no	no	no	no	no	no	no	no	no	0	46,XX[20]
HD-0490	M/74	RAEB2	2	no	p.Ser356ProfsX16	no	no	no	no	no	no	no	no	no	1	46,XY[20]
HD-0158	F/85	MDS-U	0	no	no	no	no	no	no	no	no	no	no	no	0	46,XX[20]
HD-0166	M/79	MDS-U	0	no	no	no	no	no	no	no	no	no	no	no	0	46,XY[20]
HD-0171	F/55	MDS-U	0	no	no	no	no	no	no	no	no	no	no	no	0	46,XX[20]
HD-0295	M/69	MDS-U	na	no	no	no	no	no	no	yes	no	no	no	no	1	46,XY,del(20)(q11q13)[20]
HD-0310	F/81	MDS-U	0	no	no	no	no	no	no	no	no	no	no	no	0	46,XX[20]

Table 1 : Mutations of candidate genes in a series of myelodysplastic syndromes.
hom, homozygous, MDS-U, MDS unclassified; na, not available; nc, non constitutional; RA, refractory anemia; RARS, refractory anemia with ring sideroblasts; RAEB, refractory anemia with excess of blasts; RCMD: refractory cytopenia with multilineage dysplasia. In bold, cases with acute phase in Table 2.
