# Supplementary material for: Combined mutations of ASXL1, CBL, FLT3, IDH1, IDH2, JAK2, KRAS, NPM1, NRAS, RUNX1, TET2 and WT1 genes in myelodysplastic syndromes and acute myeloid leukemias
Source: BMC Cancer. 2010 Aug 2;10:401. doi: 10.1186/1471-2407-10-401 (PMC2923633; doi:10.1186/1471-2407-10-401)
Supplement: Additional file 2 — Table S2 Mutations of candidate genes in a series of AMLs. [file 1471-2407-10-401-S2.PDF]

| Number | Sex/age | FAB subtype | Secondary to | RUNX1 (exons 1-8) | TET2 (exons 3-11) | ASXL1* (exon 12) | NPM1 (exon 12) | CBL (exons 8, 9) | FLT3 ITD/TKD* | JAK2 (V617F) | RAS* (exons 1,2) | IDH1 (exon 4) | IDH2 (exon 4) | WT1 (exons 7, 9) | Number of altered alleles | Karyotype |
|---|---|---|---|---|---|---|---|---|---|---|---|---|---|---|---|---|
| HD-0001 | M/72 | M5a | | no | p.Lys845Arg.fsX26 | no | no | no | no | no | no | no | no | no | 1 | trisomy 8 |
| HD-0003 | M/70 | M5a | | no | no | no | yes | no | no | no | no | no | no | no | 1 | normal |
| HD-0009 | F/68 | M5b | | no | p.Lys959GluX13 | no | yes | no | no | no | na | no | no | no | 2 | normal |
| HD-0010 | M/73 | M5b | | no | no | no | yes | no | no | no | no | no | no | no | 1 | normal |
| HD-0011 | F/47 | M5b | | no | no | no | yes | no | yes | no | no | no | no | no | 2 | normal |
| HD-0012 | M/21 | M5a | | no | no | no | no | no | yes | no | no | no | no | no | 1 | normal |
| HD-0016 | M/78 | M5a | | no | p.Arg1366His | no | no | no | yes | no | no | no | no | no | 2 | trisomy 8 |
| HD-0019 | M/67 | M4 | | no | no | no | yes | no | no | no | no | no | no | no | 1 | normal |
| HD-0020 | M/59 | M4 | | no | no | no | no | no | no | no | no | no | p.Arg140Gln | no | 1 | normal |
| HD-0021 | F/43 | M4 | | no | no | no | yes | no | yes | no | no | no | no | no | 2 | normal |
| HD-0026 | F/39 | M4 | | no | no | no | yes | no | no | no | yes | no | p.Arg140Gln | no | 3 | normal |
| HD-0027 | M/62 | M4 | | p.Tyr377LeuX223 | no | no | no | no | no | no | no | no | p.Arg140Gln | no | 2 | normal |
| HD-0031 | M/77 | M5a | | no | no | no | no | no | no | no | no | no | no | p.Asp299ProfsX20 | 1 | normal |
| HD-0060 | F/41 | M5a | | no | no | no | no | no | yes | no | no | no | no | no | 1 | trisomy 8 |
| HD-0097 | F/55 | M4 | | p.His105Tyr | no | no | no | no | yes | no | no | no | no | no | 2 | normal |
| HD-0099 | F/61 | M2 | | no | no | yes | no | no | no | na | yes | no | no | no | 2 | trisomy 8 |
| HD-0100 | M/78 | M2 | | no | no | no | no | no | no | no | no | no | p.Arg140Gln | no | 1 | trisomy 8 |
| HD-0101 | M/29 | M6 | | no | no | no | no | no | no | no | no | no | no | no | 0 | trisomy 8 |
| HD-0102 | F/45 | M4 | | no | no | no | yes | no | no | no | no | no | no | no | 1 | trisomy 8 |
| HD-0104 | M/79 | M2 | | p.Tyr380X | no | yes | no | no | no | no | no | no | p.Arg140Gln (nc) | no | 3 | normal |
| HD-0106 | F/59 | M5a | | no | no | no | no | no | yes | no | no | no | no | no | 1 | normal |
| HD-0107 | M/75 | M4 | | no | p.Asp1376Gly | no | yes | no | no | no | no | no | no | no | 2 | normal |
| HD-0108 | M/46 | M1 | | no | no | no | yes | no | yes | no | no | p.Arg132His | no | no | 3 | normal |
| HD-0109 | M/60 | M1 | | no | no | no | no | no | no | no | no | no | p.Arg172Lys | no | 1 | normal |
| HD-0110 | F/40 | M4 | | no | no | no | yes | no | no | no | no | p.Arg132His | no | no | 1 | normal |
| HD-0112 | F/44 | M1 | | no | no | no | yes | no | no | no | no | no | no | p.Ser313X | 2 | normal |
| HD-0113 | F/49 | M5b | | no | no | no | yes | no | yes | no | no | no | no | no | 2 | normal |
| HD-0114 | F/69 | na | | no | no | no | yes | no | yes | no | no | no | p.Arg172Lys | no | 3 | trisomy 8 |
| HD-0115 | M/71 | M2 | | no | no | no | yes | no | yes | no | no | no | p.Arg140Gln | no | 3 | trisomy 8 |
| HD-0116 | F/49 | na | | no | no | no | yes | no | no | no | no | no | no | no | 1 | normal |
| HD-0117 | F/74 | M2 | | no | no | no | yes | no | yes | no | no | no | no | no | 2 | normal |
| HD-0118 | M/69 | M5b | | no | no | no | yes | no | no | no | no | no | no | no | 1 | normal |
| HD-0119 | M/57 | M2 | | no | no | no | yes | no | no | no | no | no | no | no | 1 | normal |
| HD-0120 | M/53 | M6 | | no | no | no | no | no | no | no | no | p.Arg132Cys | no | no | 1 | normal |
| HD-0121 | F/41 | M1 | | no | no | no | yes | no | yes | no | no | no | no | no | 2 | normal |
| HD-0122 | M/64 | M2 | | no | no | no | no | no | no | no | no | no | no | no | 0 | normal |
| HD-0123 | M/83 | M1 | | no | no | no | yes | no | yes | no | no | no | p.Arg140Gln | no | 3 | normal |
| HD-0124 | F/71 | M5b | | no | no | no | no | no | yes | no | no | no | no | no | 1 | normal |
| HD-0126 | F/59 | M5b | | p.Leu313ProX287 | no | no | no | no | no | no | no | no | no | p.Ser313ValfsX4 | 2 | normal |
| HD-0128 | M/77 | M4 or 5 | | no | no | no | yes | no | no | no | no | no | p.Arg140Gln | no | 2 | normal |
| HD-0392 | F/61 | M4 | | no | no | no | yes | no | no | yes | no | no | no | no | 2 | normal |
| HD-0489 | F/60 | na | | no | no | yes | no | no | no | no | no | no | no | no | 1 | trisomy 8 |
| HD-0620 | F/71 | M5 | | no | p.Gln764Pro.fsX5 | no | no | no | no | no | no | no | no | no | 1 | normal |
| HD-0632 | F/66 | M4 | | no | no | no | yes | no | no | no | no | no | no | no | 1 | deletion 9q |
| HD-0649 | M/78 | M5b | | no | no | no | yes | no | yes | no | no | no | no | no | 2 | normal |
| HD-0693 | M/74 | M5 | | no | no | no | yes | no | yes | no | no | no | no | no | 2 | normal |
| HD-0008 | M/66 | | CMML | no | p.Asn801CysfsX14 | no | yes | no | no | no | no | no | no | no | 2 | normal |
| HD-0024 | M/61 | | CMML | no | no | no | no | no | no | no | no | no | no | no | 0 | normal |
| HD-0098 | M/77 | | MDS | no | p.Glu960SerX48 | no | no | no | no | na | no | no | no | no | 1 | trisomy 8 |
| HD-0103 | M/60 | | PMF | no | no | yes | no | no | no | no | no | no | no | no | 1 | trisomy 8 |
| HD-0105 | F/68 | | CMML | no | no | no | no | no | no | no | no | no | no | no | 0 | normal |
| HD-0111 | M/71 | | MDS | p.Ser318GlnX281 | no | yes | no | no | no | no | no | no | no | no | 2 | normal |
| HD-0125 | M/74 | | CMML | p.Arg201Gln | no | yes | no | no | no | no | no | no | no | no | 2 | normal |
| HD-0127 | F/74 | | CMML | no | no | yes | no | no | no | no | no | no | p.Arg172Lys | no | 2 | normal |
| HD-0186 | M/79 | | MDS | p.Gly170Arg | no | yes | no | no | no | no | no | no | p.Arg140Gln | no | 3 | normal |
| HD-0198 | M/73 | | CMML | break | no | yes | no | no | no | no | no | no | p.Arg172Lys (nc) | no | 3 | trisomy 8 |
| HD-0282 | M/61 | | CMML | no | no | yes | no | no | yes | no | no | no | no | no | 2 | normal |
| **HD-0304** | M/69 | | MDS (HD-0264) | no | p.Leu862SerfsX6 | yes | no | p.Glu366Lys | no | na | no | no | no | no | 3 | trisomy 11 |
| HD-0379 | F/70 | | PV | no | no | no | no | no | no | no | no | no | p.Arg140Gln | no | 1 | normal |
| HD-0381 | F/73 | | CMML | no | p.Ser792X | deletion | no | no | no | no | no | no | no | no | 2 | deletion 20q |
| HD-0402 | M/70 | | CMML | no | no | no | yes | no | yes | no | no | no | no | no | 2 | normal |
| HD-0630 | M/61 | | CMML | no | no | no | no | no | no | no | no | no | p.Arg140Gln | no | 1 | normal |
| HD-0702 | M/82 | | CMML | p.Tyr380X | no | no | no | no | no | no | yes | no | no | no | 2 | trisomy 8 |
| **HD-0790** | M/74 | | MDS (HD-0173) | p.Trp106Leu; p.Asn146MetfsX6 | no | no | no | no | no | no | no | no | no | no | 2 | normal |
