# Supplementary material for: Combined mutations of ASXL1, CBL, FLT3, IDH1, IDH2, JAK2, KRAS, NPM1, NRAS, RUNX1, TET2 and WT1 genes in myelodysplastic syndromes and acute myeloid leukemias
Source: BMC Cancer. 2010 Aug 2;10:401. doi: 10.1186/1471-2407-10-401 (PMC2923633; doi:10.1186/1471-2407-10-401)
Supplement: Additional file 4 — Table S3 Summary of results. [file 1471-2407-10-401-S4.PDF]

Table 3 : Summary of results.

**Altered genes**	**MDSs N = 65 m/t (%)**	**Secondary AMLs N = 18 m/t (%)**	**Primary AMLs N = 46 m/t (%)**	**Total N = 129 m/t (%)**
RUNX1	5/65 (7.7)	6/18 (33.3)	4/46 (8.7)	15/129 (11.6)
TET2	13/65 (20)	4/18 (22.2)	5/46 (10.9)	22/129 (17.1)
ASXL1	13/65 (20)	9/18 (50)	3/46 (6.5)	25/129 (19.4)
NPM1	0/65 (0)	2/18 (11.1)	26/46 (56.5)	28/128 (21.9)
CBL	5/65 (7.7)	1/18 (5.5)	0/46 (0)	6/129 (4.7)
FLT3	0/65 (0)	2/18 (11.1)	17/46 (37)	19/129 (14.7)
JAK2	1/65 (1.5)	0/16 (0)	1/45 (2.2)	2/126 (1.6)
RAS (N or *K)*	1/65 (1.5)	1/18 (5.6)	2/45 (4.4)	4/128 (3.1)
IDH1	2/65 (3.1)	0/18 (0)	3/46 (6.5)	5/129 (3.9)
IDH2	3/65 (4.6)	5/18 (27.8)	10/46 (21.7)	18/129 (14)
IDH1+IDH2	5/65 (7.7)	5/18 (27.8)	13/46 (28.3)	23/129 (17.8)
WT1	0/65 (0)	0/18 (0)	3/46 (6.5)	3/129 (2.3)
0 alteration	33 (50.8)	2 (11)	2 (4.3)	37/129 (28.7)
1alteration	24 (36.9)	4 (22.2)	21 (45.7)	49/129 (38)
2 alterations	7 (10.8)	9 (50)	17 (37)	33/129 (25.6)
3 alterations	2 (3.1)	3 (16.7)	6 (13)	11/129 (8.5)

Abbreviations: AML, acute myeloid leukemia; MDS, myelodysplastic syndrome; N, total number of cases; m, number of mutated cases; t, number of tested cases.
